# A New Sesquiterpene with a Novel 1*β*, 8*β*-Oxygen Bridge from *Heteropappus altaicus* (willd.) Novopokr.

**DOI:** 10.3390/molecules16010518

**Published:** 2011-01-11

**Authors:** Yi-Feng Han, Xiao-Jing Gao, Hai Huang

**Affiliations:** Department of Chemistry, Zhejiang Sci-Tech University, Hangzhou 310018, China

**Keywords:** *Heteropappus altaicus* (willd.) Novopokr., sesquiterpene, oxide bridge

## Abstract

A new guaiane-type sesquiterpene, 4*α*,7*β*-dihydroxy-10*β*H-guai-5-en-1*β*,8*β*-endoxide (**1**), was isolated from *Heteropappus altaicus* (Compositea). The structure of compound **1**, which contains exhibited a rare 1,8-oxide bridge, was established on the basis of spectroscopic data.

## 1. Introduction

*Heteropappus altaicus* (willd.) Novopokr., which belongs to the *Heteropappus* genus (Compositea, tribe Astereae), is used as traditional medicine in Tibet, and especially in Mongolia, against colds and pulmonary diseases [1,2]. The number of diterpenes, triterpenoid saponins and flavone derivatives have been identified in *H. altaicus* collected in Mongolia [3,4,5,6,7,8]. Investigation of whole plant of *H. altaicus* from Shandong Province of China, in continuation of our project to compare the chemical compositions of the same species collected from different regions, yielded a guaiane-type sesquiterpene **1**, which represents the first sesquiterpenoid of this type isolated from the *H. altaicus*. In this paper we report the isolation and structure elucidation of this compound.

## 2. Results and Discussion

Compound **1** was obtained as a yellow oil. Its molecular formula was determined as C_15_H_24_O_3_ by HREIMS, which gave a peak at *m/z* 252.1733 [M]^+^ (calcd. 252.1725) and NMR spectroscopic data (Table 1). The data indicate that the structure of **1** possesses four degrees of unsaturation. Its IR spectrum demonstrated the existence of hydroxyl (3,435 cm^-1^) and double bond (1,630 cm^-1^) functions. The ^13^C-NMR and DEPT spectra showed signals for 15 carbons, including four methyls (*δ* 25.6, 16.5, 16.2 and 16.1 ppm), three sp^3^ methylenes (*δ* 39.7, 32.8 and 30.5 ppm), two sp^3^ methines (*δ* 42.1 and 30.9 ppm), three sp^3^ quaternary oxycarbons (*δ* 88.5, 81.1 and 76.7 ppm), one sp^3^ tertiary oxycarbon (*δ* 74.4 ppm) and one double bond (*δ*153.8 and 122.7 ppm). The ^1^H-NMR spectrum of **1** displayed four methyl groups, including one tertiary methyl (*δ* 1.36, 3H, s) and three secondary methyls (*δ* 1.07, 3H, d, *J* = 6.8 Hz; 0.93, 3H, d, *J* = 6.8 Hz; 0.92, 3H, d, *J* = 6.8 Hz), one trisubstituted double bond (*δ* 5.87, 1H, s) and one oxygen-bearing methine (*δ* 4.25, 1H, d, *J* = 8.0 Hz). Thus, the tricyclic structure of **1** was revealed.

**Figure 1:**
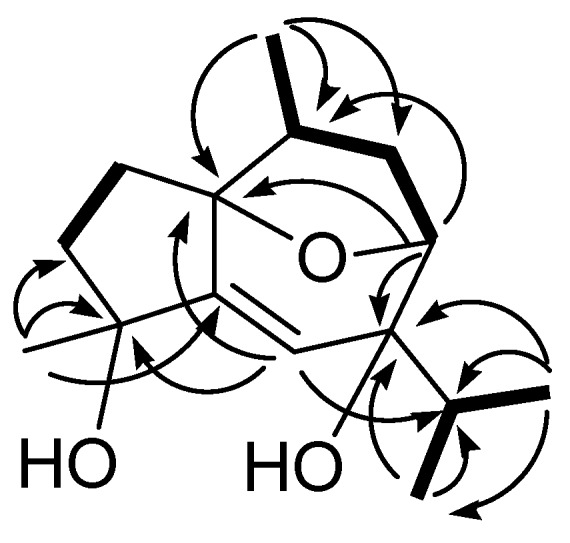
^1^H-^1^H COSY correlations (bold lines) and key HMBC correlations (H → C) of **1**.

By the analysis of ^1^H-^1^H COSY correlations (Figure 1), it was possible to establish three partial structures of consecutive proton systems extending from H_2_-2 to H_2_-3; H_3_-14 to H-10, H_2_-9 and H-8; H-11 to H_3_-12 and H_3_-13.

By further analysis of the HMBC correlations (Figure 1), the guaiane-type skeleton of **1** was established. The hydroxyl groups should be positioned at a methyl-bearing carbon C-4 and an isopropyl-bearing carbon C-7, as designated by the HMBC correlations observed from the tertiary methyl (*δ* 1.36, 3H, s) to the quaternary carbons at *δ* 76.7 (C-4) and 153.8 (C-5) and the secondary methyls (*δ* 1.07, 3H, d, *J* = 6.8 Hz; 0.92, 3H, d, *J* = 6.8 Hz) to the quaternary carbon at *δ* 74.4 (C-7), respectively. Therefore, the last one additional oxygen atom should be used to form an oxide bridge in the cycloheptene moiety of the molecule. The HMBC correlations found from the H-8 (*δ* 4.25, d, *J* = 8.0 Hz) to C-1 (*δ* 88.5, C) further confirmed the 1,8-endoxide linkage.

**Figure 2:**
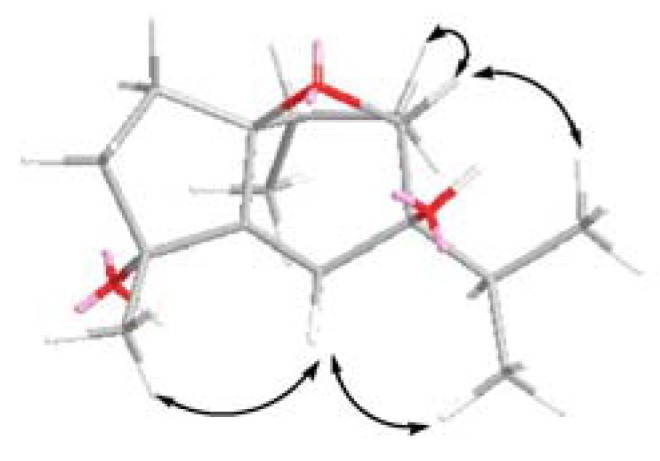
Selective NOESY correlations of **1**.

**Figure 3:**
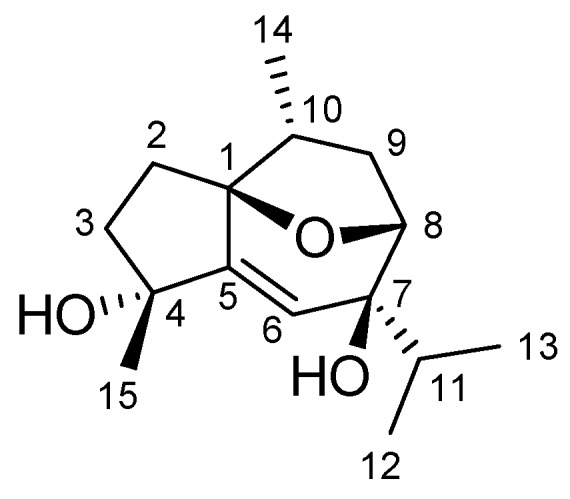
Structure of compound **1**.

In the ^1^H-NMR spectrum, the chemical shift value of H-9*α* at *δ* 2.32 was typical for guaiane-type sesquiterpenoid having *α*-methyl group at C-10 [9,10]. It was found that H_3_-15 (*δ* 1.36, 3H, s) showed a NOE interaction with H-6 (*δ* 5.87, 1H, s), suggesting the H_3_-15 should be positioned on the *β*-face which leads to a lack of NOE connectivity between H_3_-15 and H_3_-14*α*. The above observations together with the NOE correlations observed in the NOESY spectrum (Figure 2) supported the relative structure of **1** as 4*α*,7*β*-dihydroxy-10*β*H-guai-5-en-1*β*,8*β*-endoxide (Figure 3).

## 3. Experimental 

### 3.1. General

Optical rotations were measured on a Perkin-Elmer Model 341 polarimeter. IR spectra were recorded on a Nicolet NEXUS 670 FT-IR instrument, using KBr discs, over the range 400-4,000 cm^-1^. NMR spectra were measured on a Bruker AM-400 NMR spectrometer with TMS as an internal standard. High-resolution mass spectrometry experiments were obtained on a Bruker Daltonics Apex III spectrometer. Column chromatography was carried out on Si gel (200-300 mesh) and TLC on Si gel (GF254 10-40 µm), both supplied by Qingdao Marine Chemical Co.

### 3.2. Plant Material

The whole plant of *H. altaicus* (willd.) Novopokr. was collected from Taian (Shandong Province, P.R. China) and was identified by Prof. Xiaochuan Liu (School of Life Science, Zhejiang Sci-Tech University). A voucher specimen (No. 080901) has been deposited in the Department of Chemistry, Zhejiang Sci-Tech University, China.

### 3.3. Extraction and Isolation

The air-dried plant of *H. altaicus* (10 kg) was pulverized and extracted with MeOH (24.0 L) at room temperature for 7 days. The extracts were filtered and the filtrate was concentrated under reduced pressure to yield a crude methanol extract (500 g), which was suspended in H_2_O (1.0 L) and extracted with petroleum ether (boiling point 60-90 °C, 0.5 L), ethyl acetate (0.5 L) and *n*-butanol (0.5 L). The EtOAc extract (200 g) was subjected to repeated chromatography, eluting with a gradient of petroleum ether-EtOAc (20:1-0:1, v/v) and seven crude fractions (A-G) were obtained. Fraction C (2 g) was chromatographed on a silica gel column, eluting with petroleum ether-EtOAc (10:1-1:1) to give seven subfractions (C-1—C-7), of which subfraction C-4 was separated by preparative TLC developed by petroleum ether-EtOAc 7:1 to yield **1** (20 mg).

*4α,7β-Dihydroxy-10βH-guai-5-en-1β,8β-endoxide* (**1**).Yellow oil; ${\left[\alpha \right]}_{\text{D}}^{25}$ +39 (*c* 0.14 CHCl_3_); HREIMS *m/z* 252.1733 [M]^+^ (calcd. for C_15_H_24_O_3_, 252.1725); IR (KBr) *ν*_max_: 3435, 2983, 1630, 1065 cm^-1^; ^1^H- and ^13^C-NMR(CDCl_3_) data, see Table 1.

## 4. Conclusions

A new guaiane-type sesquiterpene, 4*α*,7*β*-dihydroxy-10*β*H-guai-5-en-1*β*,8*β*-endoxide (**1**), was isolated from the whole plant of *Heteropappus altaicus* (willd.) Novopokr. Compound **1** contains a rare 1,8-oxide bridge. Its structure was established on the basis of spectroscopic data. As far as we know, this is the first report of guaiane-type sesquiterpenoid isolated from the *H. altaicus* and compound **1** is also the first guaiane-type sesquiterpenoid having a 1,8-endoxide bridge.

## Figures and Tables

**Table 1:** ^1^H- and ^13^C-NMR spectra data of **1** (recorded at 400/100 MHz in CDCl_3_; *δ* in ppm, *J* in Hz).

No.	^1^H	^13^C
1	-	88.5 (C)
2	2.25 (1H, m); 1.86 (1H, m)	30.5 (CH_2_)
3	1.94 (1H, m); 1.82 (1H, m)	39.7 (CH_2_)
4	-	76.7 (C)
5	-	153.8 (C)
6	5.87 (1H, s)	122.7 (CH)
7	-	74.4 (C)
8	4.25 (1H, d, *J* = 8.0 Hz)	81.1 (CH)
9	2.32 (1H, m); 1.26 (1H, m)	32.8 (CH_2_)
10	-	42.1 (C)
11	1.84 (1H, m)	30.9 (CH)
12	0.92 (3H, d, *J* = 6.8 Hz)	16.2 (CH_3_)
13	1.07 (3H, d, *J* = 6.8 Hz)	16.5 (CH_3_)
14	0.93 (3H, d, *J* = 6.8 Hz)	16.1 (CH_3_)
15	1.36 (3H, s)	25.6 (CH_3_)
